# Psychosocial interventions for Alzheimer’s disease cognitive symptoms: a Bayesian network meta-analysis

**DOI:** 10.1186/s12877-018-0864-6

**Published:** 2018-08-07

**Authors:** Yuting Duan, Liming Lu, Juexuan Chen, Chunxiao Wu, Jielin Liang, Yan Zheng, Jinjian Wu, Peijing Rong, Chunzhi Tang

**Affiliations:** 1 Medical College of Acu-Moxi and Rehabilitation, Guangzhou University of Chinese Medicine, No.232 Waihuandong Road, Panyu District, Guangzhou, 510000 China; 2 Institute of Acupuncture and Moxibustion, China Academy of Chinese Medical Sciences, Beijing, China, 16 Nanxiaojie of Dongzhimen, Beijing, 100700 China

**Keywords:** Psychosocial intervention, Alzheimer’s disease, Cognitive symptom, Network meta-analysis

## Abstract

**Background:**

Alzheimer disease (AD) is the most common type of dementia with cognitive decline as one of the core symptoms in older adults. Numerous studies have suggested the value of psychosocial interventions to improve cognition in this population, but which one should be preferred are still matters of controversy. Consequently, we aim to compare and rank different psychosocial interventions in the management of mild to moderate AD with cognitive symptoms.

**Methods:**

We did a network meta-analysis to identify both direct and indirect evidence in relevant studies. We searched MEDLINE, EMBASE, PsycINFO through the OVID database, CENTRAL through the Cochrane Library for clinical randomized controlled trials investigating psychosocial interventions of cognitive symptoms in patients with Alzheimer disease, published up to August 31, 2017. We included trials of home-based exercise(HE), group exercise(GE), walking program(WP), reminiscence therapy(RT), art therapy(AT) or the combination of psychosocial interventions and acetylcholinesterase inhibitor (ChEIs). We extracted the relevant information from these trials with a predefined data extraction sheet and assessed the risk of bias with the Cochrane risk of bias tool. The outcomes investigated were Mini–Mental State Examination (MMSE) and compliance. We did a pair-wise meta-analysis using the fixed-effects model and then did a random-effects network meta-analysis within a Bayesian framework.

**Results:**

We deemed 10 trials eligible, including 682 patients and 11 treatments. The quality of included study was rated as low in most comparison with Cochrane tools. Treatment effects from the network meta-analysis showed WP was better than control (SMD 4.89, 95% CI -0.07 to 10.00) while cognitive training and acetylcholinesterase inhibitor (CT + ChEIs) was significantly better than the other treatments, when compared with simple ChEIs treatment, assessed by MMSE. In terms of compliance, the pair-wise meta-analysis indicated that WP and HE are better than GE and AT, while CT + ChEIs, CST + ChEIs are better than other combined interventions.

**Conclusion:**

Our study confirmed the effectiveness of psychosocial interventions for improving cognition or slowing down the progression of cognitive impairment in AD patients and recommended several interventions for clinical practice.

**Electronic supplementary material:**

The online version of this article (10.1186/s12877-018-0864-6) contains supplementary material, which is available to authorized users.

## Background

Alzheimer’s disease (AD), the most common form of dementia, is characterized by progressive synaptic loss, dysfunction, neuronal death, and vascular toxicity triggered by the deposition of pathologic inducers of lesions in the brain tissue, amyloid β peptide, and hyperphosphorylated tau protein [1]. Pharmacological interventions attempting to counteract the lesions have yet to achieve permanent successful results [2–4]. Apart from unsatisfactory efficacy, pharmacological treatments are expensive and have a series of adverse effects.

As an alternative, scientists have turned to non-pharmacological therapies, with psychosocial therapies being one of the most commonly used. In recent years, the publication of several high-quality meta-analyses, systematic reviews, and randomized controlled trials has increased the overall quality of evidence that psychosocial interventions improve or maintain cognition, function, adaptive behavior, and quality of life. For example, Epperly et al. have concluded that cognitive stimulation programs benefit the maintenance of cognitive function and improve self-reported quality of life in patients with mild to moderate Alzheimer’s disease [5]. Natasha Yuill et al. have shown that cognitive stimulation therapy is a supportive, functionally-oriented strategy aimed at enabling individuals with mild to moderate dementia to remain meaningfully engaged in their lives and surroundings [6]. The findings of Linda Clare et al. support the clinical efficacy of cognitive rehabilitation in the early stages of AD [7]. However, the 2014 American Psychiatric Association’s practice guidelines indicate that the available research has not conclusively determined whether any one intervention is more effective than any other. It also has not conclusively determined which intervention works best for which service setting, specific behavior, disease stage, or caregiver and patient profile [8].

Cognitive impairments are AD’s core clinical symptoms, and they impose the greatest burden on patients and their caregivers. Improving patients’ cognitive function can delay hospitalization, and therefore reduce the costs of national healthcare and improve both patients’ and caregivers’ well-being [9]. In order to offer high-quality evidence for clinical decisions, we performed a Bayesian network meta-analysis to compare and rank different psychosocial interventions in the management of cognitive symptoms in patients with AD.

## Methods

This study was conducted in accordance with the Cochrane Handbook for the Systematic Review of Interventions (see details at http://training.cochrane.org/handbook) and the Preferred Reporting Items for Systematic Review and Meta-Analyses [10]. Included studies were classified according to the type of psychosocial intervention.

### Search strategy

For the two network meta-analyses, we searched MEDLINE, EMBASE and PsycINFO through the OVID database, and searched CENTRAL through the Cochrane Library. We searched studies published from inception to August 31, 2017, and compared any psychosocial interventions for cognitive symptoms in patients with AD (Additional file 1).

### Study selection

Inclusion criteria and exclusion criteria are summarized in Table 1.

**Table 1:** Eligibility Criteria PICOS

	Inclusion Criteria	Exclusion Criteria
Participants	Meet the diagnosis of National Institute of Neurological and Communication Disorders and Stroke/Alzheimer’s Disease and Related Disorders Association (NINCDS/ADRDA) criteria or The Diagnostic and Statistical Manual of Mental Disorders (DSM).	Mild cognitive impairment or other types of non-AD dementia; familial AD initiated before 50 y old or related to other genetic diseases.
Interventions	Any type of psychosocial interventions or combination of psychosocial interventions and ChEIs.	
Comparisons	ChEIs (positive control); usual care (normal control)	
Outcomes	Primary outcomes: MMSE; Secondary outcomes: Compliance	
Study design	Randomized controlled trials; sample size>10/arm.	

We included the following psychosocial interventions with usual care as the control: home-based exercise (HE), group exercise (GE), walking programs (WP), reminiscence therapy (RT), and art therapy (AT). We also included the combination of psychosocial interventions and ChEIs with ChEIs as a positive control: the combination of cognitive stimulation treatment and acetylcholinesterase inhibitor (CST + ChEIs), the combination of mindfulness-based Alzheimer’s stimulation and acetylcholinesterase inhibitor (MBAS+ChEIs), the combination of progressive muscle relaxation and acetylcholinesterase inhibitor (PMR + ChEIs), and the combination of cognitive training and acetylcholinesterase inhibitor (CT + ChEIs).

### Data extraction and quality assessment

Three investigators (YTD, LLM, JXC) independently selected the studies. The review of the main reports and supplementary materials, the extractions of the relevant information from the included trials with a predetermined data extraction sheet, and the assessments of the risk of bias with the Cochrane risk of bias tool were independently performed by three investigators (CXW, JLL, YZ). Any disagreements were resolved through discussion. When they did not reach a consensus, the final decision about each question was made by other investigators within the review team (JJW, CZT, PJR).

We evaluated the quality of the included studies with the Cochrane Collaboration Recommendations assessment tool. The tool for assessing 7 domains, including random sequence generation, allocation concealment, blinding of participants and personnel, blinding (or masking) of outcomes assessors, incomplete outcome data, selective reporting and other bias is described in the Cochrane Handbook for Systematic Reviews of Interventions (see details at http://training.cochrane.org/handbook). Based on these items, studies in which the key domains were all low-risk were considered low-risk, while the remainder were deemed high-risk or unclear-risk, depending on the number of key domains of high or unclear risk.

### Statistical analysis

A network meta-analysis with a Bayesian framework with Aggregate Data Drug Information System (ADDIS, version 1.16.8) was conducted to assess the cognitive outcomes of psychosocial interventions. This software is based on the Bayesian framework and the Markov chain Monte Carlo method which can evaluate a priori and process research data. We used a random-effects model to analyze the effect sizes in this study. The effect sizes for continuous outcomes were the mean difference (MD). Consistency and inconsistency were the two models used to estimate the effect size in ADDIS. A consistency assessment drew conclusions on effect sizes of the included interventions and estimated the ranking probabilities for all the interventions. The consistency test was judged by node-splitting analysis and an inconsistence model. When the *p*-value of the node-splitting analysis was greater than 0.05, a consistency mode was selected [11]. Otherwise, an inconsistency model was used. Potential scale reduction factor (PSRF) was used to evaluate the convergence of the model. The closer the PSRF value was to 1, the better the convergence. The convergence of the model was still acceptable if the PSRF value was less than 1.2. For each intervention, we estimated the ranking probabilities for each treatment at each possible rank.

We ran pair-wise meta-analyses to compare the compliance of different psychosocial therapies because the data included in our study were insufficient for statistical analysis of the network meta-analysis. We conducted the pair-wise meta-analysis with the fixed-effects model with Review Manager (RevMan, v 5.3). The odds ratio (OR) was calculated for dichotomous outcomes (compliance), with 95% credible intervals (CI). We assessed statistical heterogeneity in the pair-wise comparison with an I^2^ statistic and the *p*-value.

## Results

### Study identification and selection

**Fig. 1:**
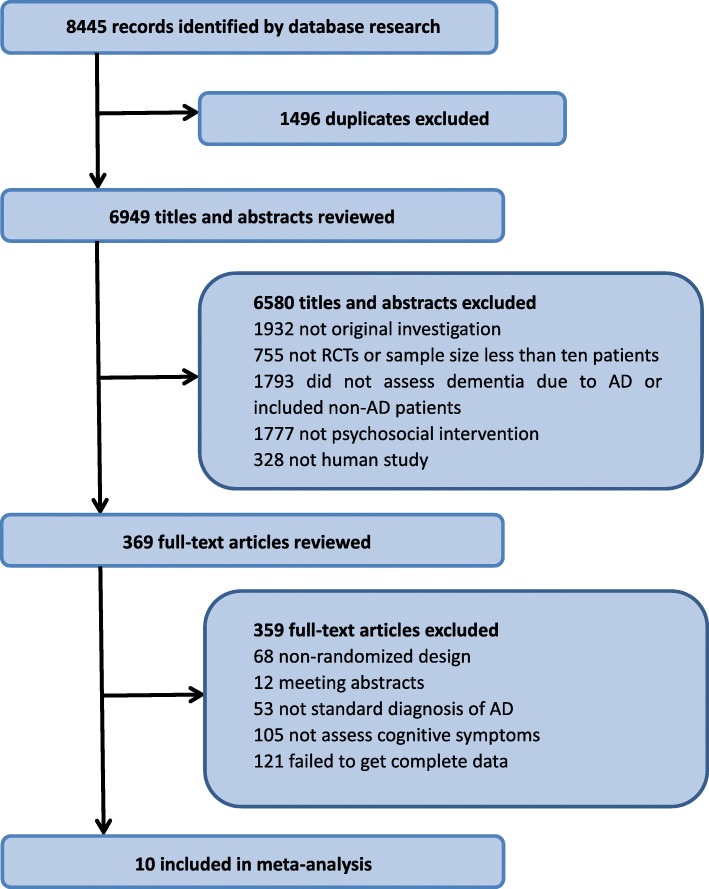
Study Selection

In total, 8445 citations published between 1981 and August 31, 2017 was identified by the search. After removing duplicates and unrelated articles, 10 articles describing 11 RCTs including 682 patients were eligible for further quantitative analyses. A flow chart of the specific screening procedures is shown in Fig. 1. The baseline characteristics of the studies were also extracted (Table 2).

**Table 2:** Baseline of Included studies

Year	First author	Study design	Principle health problem	Mean age in years	SEX	MMSE Inclusion criteria	Final sample size	Intervention	Duration	Control	Main outcomes	Main findings	Risk of bias
2011	Hideyuki Hattori [20]	RCT	mild AD	74.32	M/F (18/21)	20–24	39	AT	12 weeks	Usual care	QOL; ADL; MMSE,WMS-R; GDS,	These results suggested improvement in at least the vitality and the QOL of patients with mild Alzheimer’s disease after art therapy.	unclear
2016	Domingo J [21]	Double-blind RCT	AD	No mention	M/F (54/66)	≥18	120	MBAS+ ChEIs; CST+ ChEIs; PMR + ChEIs	2 years	ChEIs	MMSE; CAMCOG	The mindfulness group showed significant scores compared with the control and muscle relaxation groups. Group cognitive stimulation evolved better than the control group but not better than the muscle relaxation group.	low
2010	Yi-Xuan Niu [22]	rater-blind RCT	mild to moderate AD	79.85	M/F (25/7)	10–24	32	CST + ChEIs	10 weeks	ChEIs	NPI; MMSE	The study showed that cognitive stimulation therapy has significant efficacy in lowering apathy and depression symptomatology and in the Mini Mental State Examination in patients with mild to moderate AD.	low
2006	L Ta′rraga [23]	single-blind, pilot RCT	AD	76.7	F (84.78)	18–24	43	CST + ChEIs	24 weeks	ChEIs	ADAS-Cog;MMSE	Cognitive stimulation treatment improved cognition in patients who were treated with a stable dose of ChEI, compared with those who were treated only with ChEIs.	unclear
2015	Grazia D’Onofrio [24]	single-blind RCT	AD	78.19	M/F (42/48)	≥10	90	CST + ChEIs	6-month	ChEIs	MMSE;CDR;HAMD	The study showed that the integrated treatment of RTP with CS in AD patients for 6 months improved significantly cognition, depressive and neuropsychiatric symptoms, fuctional status, and mortality risk in comparison with a group of AD patients receiving only RTP.	low
2013	Susanna Bergamaschi [25]	single-blind RCT	AD	77.96	no mention	18–24	32	CT + ChEIs	1 year	ChEIs	MMSE; MODA; CSDD; ADL	The study found that patients who participated in the CT intervention showed improvement in several cognitive measures and did not experience any decline on neuropsychological tests or in activities of daily living.	low
2016	Hannu Kautiainen [26]	multicenter, RCT	AD and their spousal caregivers	77.83	F (38.57%)	no mention	161	HE; GE	1 year	drug naive	CDT; VF; CDR,MMSE	The study found that participation in a long-term, customized home exercise program may have some effect, albeit modest, on executive function in individuals with AD	unclear
2016	Guler Duru Asiret [27]	RCT	mild and moderate AD	82.05	M/F (20/41)	10–24	62	RT	12 weeks	Not reported	GDS; MMSE; ADL	We found reminiscence therapy to have a beneficial effect on the cognitive status and depression in institutionalized patients with mild to moderate AD in our study.	unclear
2013	Peter Van Bogaert RN [28]	a pilot RCT	older adults with probable AD	84	F (82.9%)	no mention	82	RT	4 weeks	Not reported	MMSE,FAB,NPI,CSDD,GDS-30	The pilot study results showed positive effects associated with individual thematically-based reminiscence on well-being such as depressive symptoms and cognition of participants.	high
2011	Massimo Venturelli [29]	RCT	the later stages of Alzheimer’s disease	84	M (14.28%)	5–15	21	WP	24 weeks	Not reported	ADL; MMSE	The WG showed significant improvement in the 6WT and ADLs, while WG decreased in MMSE.	low

### Quality assessment of included studies

We evaluated the quality of included studies with the Cochrane Collaboration Recommendations assessment tools [12]. Among 10 trials, 8 studies (80%) described a random component in the sequence generation process such as a computer-generated random number or a random number table. The others did not permit judgment of ‘low risk’ or ‘high risk’ due to insufficient information about the sequence generation process. Allocation concealment was performed using an appropriately sealed method in 70% (7) of the studies, while 30% (3) either did not describe concrete methods or used an inappropriate allocation concealment method. In performance bias, 70% (7) of the included trials reported the methods of blinding for both participants and personnel. In detection bias, 30% (3) of the outcome assessors in the studies either could not be blinded or were unclear. In attrition bias, 9 studies were deemed to have low-risk outcome data (i.e. reported dropout rates within the range of statistical estimations, provided detailed explanations of dropout rates or performed intention-to-treat analysis). 1 study did not provide adequate information to judge the risk of missing data. Other risks were unclear due to insufficient information in 5 studies. Overall, 1 study was considered high risk and 5 were considered low risk, while 4 were considered unclear risk. A detailed quality assessment is presented in Additional files 2 and 3.

### Meta-analyses

**Fig. 2:**
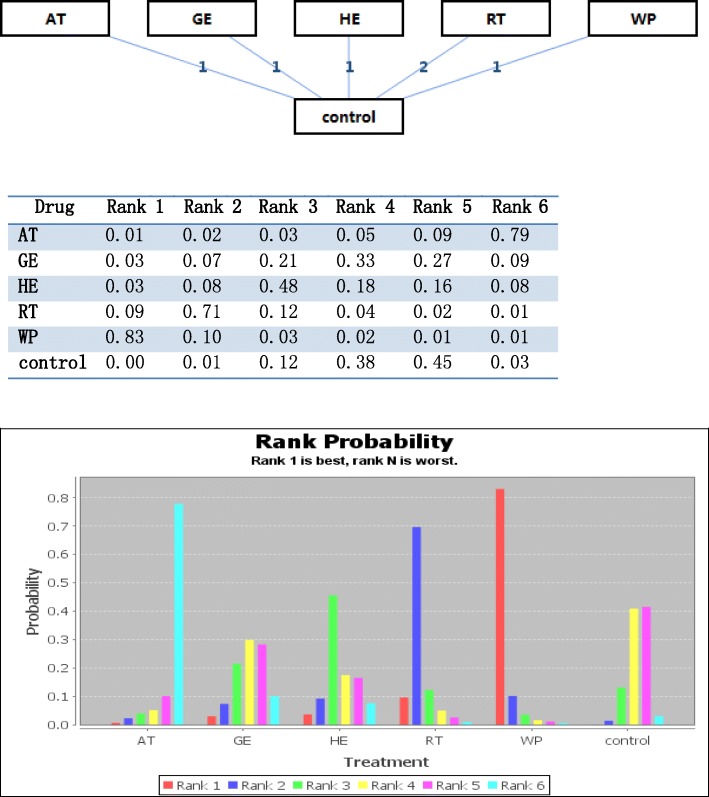
Rank Probability of Cognitive Effect of the Psychosocial Interventions. *AT* art therapy, *GE* group exercise, *HE* home-based exercise, *RT* reminiscence, therapy, *WP* walking program. Control = usual care

A network meta-analysis was performed to compare and rank the included psychosocial interventions which used usual care as their control. The network of eligible comparisons for efficacy consisted of 6 studies and 5 treatments (2 arms of RT; 1 arm of AT, GE, HE, WP; 6 arms of control). The specific network is presented in Fig. 2a. The consistency model was selected for the subsequent network analyses. Meanwhile, the inconsistency model was used to test consistency. The results of the network meta-analysis for the primary outcomes are presented as a league table in Table 3. In terms of efficacy, WP was better than the control (SMD 4.98, 95% CI -0.07 to 10.00). This was the best psychosocial intervention for improving cognitive symptoms assessed by the Mini–Mental State Examination (MMSE). The results indicated that WP was significantly more effective than the other treatments in our study. The second and third most effective interventions were RT and HE. The ranking probability of treatments is presented in Fig. 2b and c

**Table 3:** Network Meta-Analysis of Cognitive Effect of Psychosocial Interventions

Consistency Model of Psychosocial Interventions					
AT	2.48 (−4.43, 9.24)	2.86 (− 4.16, 9.50)	4.84 (− 1.29, 10.78)	7.29 (− 0.04, 14.07)	2.29 (−2.81, 7.27)
− 2.48 (− 9.24, 4.43)	GE	0.39 (− 6.67, 6.84)	2.37 (− 3.44, 8.07)	4.78 (− 2.48, 11.61)	− 0.16 (− 4.99, 4.46)
− 2.86 (− 9.50, 4.16)	−0.39 (− 6.84, 6.67)	HE	2.00 (− 3.76, 7.77)	4.37 (− 2.14, 11.10)	− 0.55 (− 5.17, 4.14)
− 4.84 (− 10.78, 1.29)	− 2.37 (− 8.07, 3.44)	−2.00 (−7.77, 3.76)	RT	2.38 (− 3.41, 8.34)	− 2.53 (− 5.89, 0.69)
− 7.29 (− 14.07, 0.04)	−4.78 (− 11.61, 2.48)	−4.37 (− 11.10, 2.14)	−2.38 (− 8.34, 3.41)	WP	−4.89 (− 10.00, 0.07)
− 2.29 (− 7.27, 2.81)	0.16 (− 4.46, 4.99)	0.55 (− 4.14, 5.17)	2.53 (− 0.69, 5.89)	4.89 (− 0.07, 10.00)	control
Inconsistency Model of Psychosocial Interventions					
AT	2.44 (− 4.28, 9.85)	2.81 (− 3.92, 9.76)	4.79 (− 1.16, 10.97)	7.24 (0.38, 14.65)	2.26 (− 2.56, 7.52)
−2.44 (− 9.85, 4.28)	GE	0.39 (− 6.56, 6.68)	2.34 (−3.71, 7.94)	4.74 (− 2.15, 11.43)	− 0.16 (− 4.90, 4.38)
− 2.81 (− 9.76, 3.92)	−0.39 (− 6.68, 6.56)	HE	1.99 (− 3.98, 7.67)	4.39 (− 2.58, 11.46)	−0.54 (− 5.39, 4.44)
− 4.79 (− 10.97, 1.16)	− 2.34 (− 7.94, 3.71)	−1.99 (− 7.67, 3.98)	RT	2.37 (−3.54, 8.61)	− 2.53 (− 5.91, 1.06)
− 7.24 (− 14.65, − 0.38)	− 4.74 (− 11.43, 2.15)	− 4.39 (− 11.46, 2.58)	−2.37 (− 8.61, 3.54)	WP	− 4.94 (− 9.90, 0.04)
− 2.26 (− 7.52, 2.56)	0.16 (− 4.38, 4.90)	0.54 (− 4.44, 5.39)	2.53 (− 1.06, 5.91)	4.94 (− 0.04, 9.90)	control

**Fig. 3:**
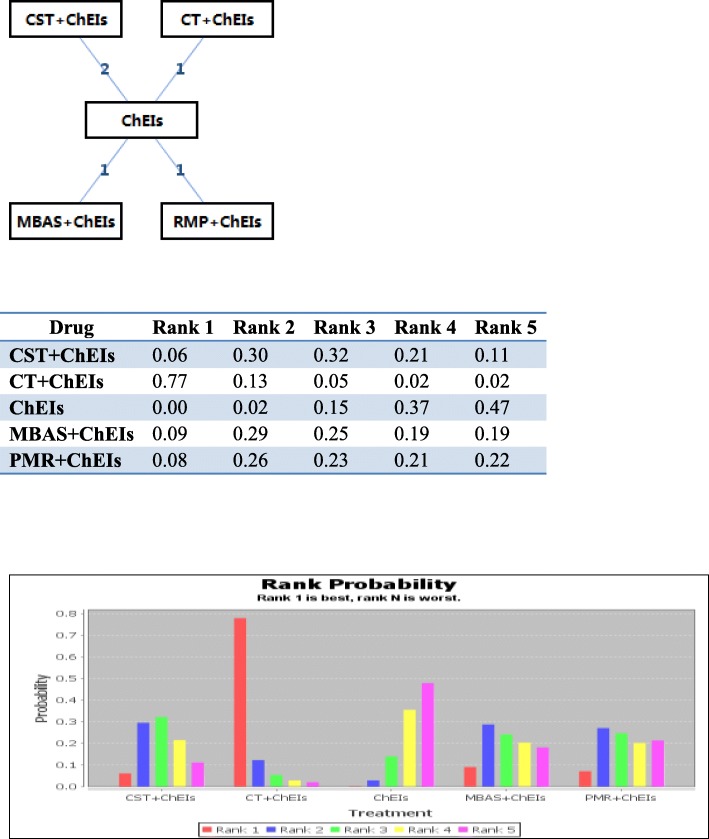
Rank Probability of Cognitive Effect of the Combination of Psychosocial Interventions and ChEIs. CST + ChEIs = the combination of cognitive stimulation treatment and acetylcholinesterase inhibitor; CT + ChEIs = the combination of cognitive training and acetylcholinesterase inhibitor; ChEIs = acetylcholinesterase inhibitor; MBAS+ChEIs = the combination of progressive muscle relaxation and acetylcholinesterase inhibitor; PMR + ChEIs = the combination of progressive muscle relaxation and acetylcholinesterase inhibitor

Consisting of 5 studies and 5 treatments (2 arms of CST + ChEIs; 1 arm of CT + ChEIs, MBAS+ChEIs, RMP + ChEIs; 5 arms of ChEIs), another network meta-analysis was run to assess the effectiveness of the combination of psychosocial interventions with ChEIs and simple ChEIs treatment. The specific network is presented in Fig. 3a. In this subsequent network analysis, we used a consistency model, while an inconsistency model was used to test consistency. The results of the network meta-analyses for the primary outcomes are presented as a league table in Table 4. Note that the curative effect, CT + ChEIs, the best combined intervention for improving cognitive symptoms assessed by MMSE, was better than the control (SMD 6.27, 95%CI -1.05 to 13.44). These results suggested that CT + ChEIs was significantly more effective than the other treatments. The second and third most effective interventions were CST + ChEIs and MBAS+ChEIs. The three most effective combined interventions were better than the ChEIs. The ranking probability of treatments is presented in Fig. 3b and c.

**Table 4:** Network Meta-Analysis of Cognitive Effect of the Combination of Psychosocial Interventions and ChEIs

Consistency Model of Psychosocial Interventions				
CST + ChEIs	4.51 (− 4.51, 13.02)	− 1.73 (− 7.07, 3.33)	− 0.09 (− 9.03, 8.71)	−0.29 (− 9.56, 8.33)
−4.51 (− 13.02, 4.51)	CT + ChEIs	−6.27 (− 13.44, 1.05)	− 4.60 (− 14.92, 5.85)	−4.82 (− 15.10, 5.75)
1.73 (− 3.33, 7.07)	6.27 (−1.05, 13.44)	ChEIs	1.70 (− 5.64, 9.04)	1.43 (− 6.00, 8.76)
0.09 (− 8.71, 9.03)	4.60 (− 5.85, 14.92)	− 1.70 (− 9.04, 5.64)	MBAS + ChEIs	−0.26 (− 11.00, 10.26)
0.29 (− 8.33, 9.56)	4.82 (− 5.75, 15.10)	−1.43 (− 8.76, 6.00)	0.26 (− 10.26, 11.00)	PMR + ChEIs
Inconsistency Model of Psychosocial Interventions				
CST + ChEIs	4.58 (− 4.49, 13.47)	−1.74 (− 6.90, 3.37)	− 0.12 (− 8.89, 8.93)	− 0.23 (− 9.24, 8.39)
− 4.58 (− 13.47, 4.49)	CT + ChEIs	−6.36 (− 13.47, 0.90)	− 4.73 (− 14.64, 5.67)	−4.90 (− 14.98, 5.24)
1.74 (− 3.37, 6.90)	6.36 (− 0.90, 13.47)	ChEIs	1.66 (− 5.65, 8.86)	1.48 (− 5.74, 8.52)
0.12 (− 8.93, 8.89)	4.73 (− 5.67, 14.64)	−1.66 (− 8.86, 5.65)	MBAS + ChEIs	−0.18 (− 10.27, 9.63)
0.23 (− 8.39, 9.24)	4.90 (− 5.24, 14.98)	−1.48 (− 8.52, 5.74)	0.18 (− 9.63, 10.27)	PMR + ChEIs

Additional file 4 shows the results of pair-wise meta-analyses of compliance for each intervention. The included studies which were not analyzed did not have missing data due to patient non-compliance. We can conclude that patients’ compliance with RT, WP and HE are better than GE and AT, and that patients’ compliance with CT + ChEIs, CST + ChEIs were better than those of MBAS+ChEIs, RMP + ChEIs and ChEIs.

## Discussion

These findings regarding the comprehensive network meta-analysis represent the most comprehensive synthesis of data for currently available psychosocial AD treatments including the combined treatments of psychosocial therapies and ChEIs. We found that the cognitive effects of WP, RT, HE and GE are better than the usual care. The combination of the following psychosocial interventions and ChEIs are more effective than ChEIs: CT + ChEIs, CST + ChEIs, MBAS+ChEIs and PMR + ChEIs.

Our study supplements the recommendations of existing guidelines and identifies specific psychosocial interventions with better effects. When only using psychosocial interventions for AD patients, WP and RT showed better efficacy in the management of cognitive symptoms. WP is a physical activity, which is a type of stimulation-oriented intervention. Several clinical investigations have shown its cognitive benefits for older individuals who are healthy, but with mild cognitive impairment or dementia [13–16]. When WP is applied, the adverse reactions of its application should be taken into consideration. For personalized needs, the frequency and intensity of WP should be targeted adjustment. Reminiscence therapy is defined as emotion-oriented interventions in which an individual remembers a past event, verbally or nonverbally, alone or with a group. Reminiscence therapy is another commonly used non-pharmacological application for AD and other types of dementia which benefits cognition and mental health [17–19].

We also compared and ranked the combination of psychosocial interventions and ChEIs and single used ChEIs, which are the most common used pharmacological interventions. Although there was not enough evidence to prove that psychosocial interventions were better than pharmacological interventions, our study found that the combination of psychosocial interventions and pharmacological interventions was better than treatment with single drugs. Among the included treatments, CT programs combined with pharmacological treatments that could protect patients from functional deterioration by slowing progressive decline showed the best efficacy for cognitive impairments in AD patients. This revealed that clinical practitioners can try combined therapy, rather than only using drugs.

Evidence indicates that pharmacological treatments for AD can benefit patients, but important side effects have led to the development of non-pharmacological interventions and their widespread use. We confirmed that the efficacy of psychosocial therapies and combined interventions are better than pharmacological therapies used alone. However, the long-term benefits and the potential for translating these approaches into practice remain uncertain. Meanwhile, the cost-effectiveness also needs to be evaluated with regards to the high education fees for caregivers, as well as the economic burdens for society.

There are several limitations to this review and the data used for the meta-analysis. First, the quality of the included studies was not optimal. When evaluating these studies, we found that many lacked details on randomization or blinding, especially for psychosocial interventions that were difficult to blind. Additionally, several studies had high dropout rates, due inevitably to the length of the trials. Second, although we evaluated the studies according to the tool, any evaluation of bias is subjective. There is no quantitative index that can evaluate only artificial risk of bias. Third, because we used strict inclusion and exclusion criteria, the amount of included studies was less. This may have influenced the strength of the evidence. Fourth, there was no unified index for the classification of intervention methods. Therefore, we categorized them by the descriptions in the literature.

## Conclusions

Our study confirmed the effectiveness of psychosocial interventions for improving cognition or slowing the progression of cognitive impairment in AD patients. It also recommended several interventions for clinical practice. Future research should be conducted to confirm the impact of psychosocial therapy on other AD symptoms and additional high-quality RCTs should be performed to provide more powerful evidence. Furthermore, the cost-effectiveness of psychosocial interventions will have to be analyzed.

## Additional files

Additional file 1: Search Strategy. (DOCX 20 kb)

Additional file 2: Risk of Bias Graph. (DOCX 31 kb)

Additional file 3: Risk of Bias Summary. (DOCX 25 kb)

Additional file 4: Pair-wise meta-analysis of Compliance for Each Intervention. (DOCX 218 kb)

## Abbreviations

AD: Alzheimer disease
ADDIS: Aggregate Data Drug Information System
AT: Art therapy
ChEIs: Acetylcholinesterase inhibitor
CI: Credible intervals
CST + ChEIs: The combination of cognitive stimulation treatment and acetylcholinesterase inhibitor
CT + ChEIs: And the combination of cognitive training and acetylcholinesterase inhibitor
GE: Group exercise
HE: Home-based exercise
MBAS+ChEIs: the combination of mindfulness-based Alzheimer’s stimulation and acetylcholinesterase inhibitor
MMSE: Mini–Mental State Examination
OR: Odds ratio
PMR + ChEIs: The combination of progressive muscle relaxation and acetylcholinesterase inhibitor
PSRF: Potential scale reduction factor
RevMan: Review Manager
RT: Reminiscence therapy
WP: Walking program
