# Supplementary material for: Psychosocial interventions for Alzheimer’s disease cognitive symptoms: a Bayesian network meta-analysis
Source: BMC Geriatr. 2018 Aug 7;18:175. doi: 10.1186/s12877-018-0864-6 (PMC6081912; doi:10.1186/s12877-018-0864-6)
Supplement: Supplementary file 1 — Search Strategy. (DOCX 20 kb) [file 12877_2018_864_MOESM1_ESM.docx]

# Search strategy

**MEDLINE (OVID) search strategy, August 31, 2017**

1. exp Dementia.mp.
2. (Dementias OR Amentia OR Amentias OR Senile Paranoid Dementia OR Dementias, Senile Paranoid OR Paranoid Dementia, Senile OR Paranoid Dementias, Senile OR Senile Paranoid Dementias OR Familial Dementia OR Dementia, Familial

OR Dementias, Familial OR Familial Dementias) .tw.

1. 1 OR 2
2. exp Alzheimer Disease.mp.
3. (Disease, Alzheimer OR Alzheimer Sclerosis OR Sclerosis, Alzheimer OR Alzheimer Syndrome OR Syndrome, Alzheimer OR Alzheimer Dementia OR Dementia, Alzheimer OR Alzheimer-Type Dementia OR Alzheimer Type Dementia OR Dementia, Alzheimer-Type OR Primary Senile Degenerative Dementia OR Dementia, Senile OR Senile Dementia OR Dementia, Alzheimer Type OR Alzheimer Type Dementia OR Senile Dementia, Alzheimer Type OR Alzheimer Type Senile Dementia OR Dementia, Primary Senile Degenerative OR Alzheimer's Disease OR Disease, Alzheimer's OR Acute Confusional Senile Dementia OR Senile Dementia, Acute Confusional OR Dementia, Presenile OR Presenile Dementia OR Alzheimer Disease, Late Onset OR Late Onset Alzheimer Disease OR Alzheimer's Disease, Focal Onset OR Focal Onset Alzheimer's Disease OR Familial Alzheimer Disease OR Alzheimer Disease, Early Onset Early Onset Alzheimer Disease OR Presenile Alzheimer Dementia).tw.
4. 4 OR 5
5. exp Drug Therapy.mp.
6. (Therapy, Drug OR Drug Therapies OR Therapies, Drug OR Chemotherapy OR Chemotherapies OR Pharmacotherapy OR Pharmacotherapies).tw.
7. 7 OR 8
8. exp Psychology,Social.mp.
9. exp Psychotherapy.mp.
10. (incentive*OR voucher OR psychotherap* OR psychosocial* OR behaviour therapy OR behavior therapy OR reinforcement OR motivation* OR contingent* OR advice OR biofeedback OR community OR stimulation OR education* OR brief intervention OR early intervention OR minimal intervention OR counseling OR counsel* OR cognitive therapy OR family therapy OR social skill OR stress management training OR supportive expressive therapy OR neurobehavioral* OR coping skill* OR "self-control training").tw.

13. 10 OR 11 OR 12

1. exp Complementary Therapies.mp.
2. (Therapies, Complementary OR Therapy, Complementary OR Complementary Medicine OR Medicine, Complementary OR Alternative Medicine OR Medicine, Alternative OR Alternative Therapies OR Therapies, Alternative OR Therapy, Alternative).tw.

16. 14 OR 15

1. Randomized controlled trial.pt.
2. controlled clinical trial.pt.
3. randomized.ti,ab.
4. placebo.ti,ab.
5. randomly.ti,ab.
6. trial.ti,ab.
7. groups.ti,ab.

24. OR/17-23

25. 9 OR 13 OR 16

26. 3 AND 6 AND 24 AND 25

# EMBASE (OVID) search strategy, August 31, 2017

1. exp Dementia.mp.
2. (Dementias OR Amentia OR Amentias OR Senile Paranoid Dementia OR Dementias, Senile Paranoid OR Paranoid Dementia, Senile OR Paranoid Dementias, Senile OR Senile Paranoid Dementias OR Familial Dementia OR Dementia, Familial

OR Dementias, Familial OR Familial Dementias) .tw.

1. 1 OR 2
2. exp Alzheimer Disease.mp.
3. (Disease, Alzheimer OR Alzheimer Sclerosis OR Sclerosis, Alzheimer OR Alzheimer Syndrome OR Syndrome, Alzheimer OR Alzheimer Dementia OR Dementia, Alzheimer OR Alzheimer-Type Dementia OR Alzheimer Type Dementia OR Dementia, Alzheimer-Type OR Primary Senile Degenerative Dementia OR Dementia, Senile OR Senile Dementia OR Dementia, Alzheimer Type OR Alzheimer Type Dementia OR Senile Dementia, Alzheimer Type OR Alzheimer Type Senile Dementia OR Dementia, Primary Senile Degenerative OR Alzheimer's Disease OR Disease, Alzheimer's OR Acute Confusional Senile Dementia OR Senile Dementia, Acute Confusional OR Dementia, Presenile OR Presenile Dementia OR Alzheimer Disease, Late Onset OR Late Onset Alzheimer Disease OR Alzheimer's Disease, Focal Onset OR Focal Onset Alzheimer's Disease OR Familial Alzheimer Disease OR Alzheimer Disease, Early Onset Early Onset Alzheimer Disease OR Presenile Alzheimer Dementia).tw.
4. 4 OR 5
5. exp Drug Therapy.mp.
6. (Therapy, Drug OR Drug Therapies OR Therapies, Drug OR Chemotherapy OR Chemotherapies OR Pharmacotherapy OR Pharmacotherapies).tw.
7. 7 OR 8
8. exp Psychology,Social.mp.
9. exp Psychotherapy.mp.
10. (incentive*OR voucher OR psychotherap* OR psychosocial* OR behaviour therapy OR behavior therapy OR reinforcement OR motivation* OR contingent* OR advice OR biofeedback OR community OR stimulation OR education* OR brief intervention OR early intervention OR minimal intervention OR counseling OR counsel* OR cognitive therapy OR family therapy OR social skill OR stress management training OR supportive expressive therapy OR neurobehavioral* OR coping skill* OR "self-control training").tw.

13. 10 OR 11 OR 12

1. exp Complementary Therapies.mp.
2. (Therapies, Complementary OR Therapy, Complementary OR Complementary Medicine OR Medicine, Complementary OR Alternative Medicine OR Medicine, Alternative OR Alternative Therapies OR Therapies, Alternative OR Therapy, Alternative).tw.

16. 14 OR 15

1. Randomized controlled trial.pt.
2. controlled clinical trial.pt.
3. randomized.ti,ab.
4. placebo.ti,ab.
5. randomly.ti,ab.
6. trial.ti,ab.
7. groups.ti,ab.

24. OR/17-23

25. 9 OR 13 OR 16

26. 3 AND 6 AND 24 AND 25

# PsycINFO (OVID) search strategy, August 31,2017

1. exp Dementia.mp.
2. (Dementias OR Amentia OR Amentias OR Senile Paranoid Dementia OR Dementias, Senile Paranoid OR Paranoid Dementia, Senile OR Paranoid Dementias, Senile OR Senile Paranoid Dementias OR Familial Dementia OR Dementia, Familial

OR Dementias, Familial OR Familial Dementias) .tw.

1. 1 OR 2
2. exp Alzheimer Disease.mp.
3. (Disease, Alzheimer OR Alzheimer Sclerosis OR Sclerosis, Alzheimer OR Alzheimer Syndrome OR Syndrome, Alzheimer OR Alzheimer Dementia OR Dementia, Alzheimer OR Alzheimer-Type Dementia OR Alzheimer Type Dementia OR Dementia, Alzheimer-Type OR Primary Senile Degenerative Dementia OR Dementia, Senile OR Senile Dementia OR Dementia, Alzheimer Type OR Alzheimer Type Dementia OR Senile Dementia, Alzheimer Type OR Alzheimer Type Senile Dementia OR Dementia, Primary Senile Degenerative OR Alzheimer's Disease OR Disease, Alzheimer's OR Acute Confusional Senile Dementia OR Senile Dementia, Acute Confusional OR Dementia, Presenile OR Presenile Dementia OR Alzheimer Disease, Late Onset OR Late Onset Alzheimer Disease OR Alzheimer's Disease, Focal Onset OR Focal Onset Alzheimer's Disease OR Familial Alzheimer Disease OR Alzheimer Disease, Early Onset Early Onset Alzheimer Disease OR Presenile Alzheimer Dementia).tw.
4. 4 OR 5
5. exp Drug Therapy.mp.
6. (Therapy, Drug OR Drug Therapies OR Therapies, Drug OR Chemotherapy OR Chemotherapies OR Pharmacotherapy OR Pharmacotherapies).tw.
7. 7 OR 8
8. exp Psychology,Social.mp.
9. exp Psychotherapy.mp.
10. (incentive*OR voucher OR psychotherap* OR psychosocial* OR behaviour therapy OR behavior therapy OR reinforcement OR motivation* OR contingent* OR advice OR biofeedback OR community OR stimulation OR education* OR brief intervention OR early intervention OR minimal intervention OR counseling OR counsel* OR cognitive therapy OR family therapy OR social skill OR stress management training OR supportive expressive therapy OR neurobehavioral* OR coping skill* OR "self-control training").tw.

13. 10 OR 11 OR 12

1. exp Complementary Therapies.mp.
2. (Therapies, Complementary OR Therapy, Complementary OR Complementary Medicine OR Medicine, Complementary OR Alternative Medicine OR Medicine, Alternative OR Alternative Therapies OR Therapies, Alternative OR Therapy, Alternative).tw.

16. 14 OR 15

1. Randomized controlled trial.pt.
2. controlled clinical trial.pt.
3. randomized.ti,ab.
4. placebo.ti,ab.
5. randomly.ti,ab.
6. trial.ti,ab.
7. groups.ti,ab.

24. OR/17-23

25. 9 OR 13 OR 16

26. 3 AND 6 AND 24 AND 25

# CENTRAL (Cochrane Library) search strategy, August 31, 2017

1. MeSH descriptor: [Dementia] explode all trees
2. (Dementias or Amentia or Amentias or Senile Paranoid Dementia or Dementias, Senile Paranoid or Paranoid Dementia, Senile or Paranoid Dementias, Senile or Senile Paranoid Dementias or Familial Dementia or Dementia, Familial or Dementias, Familial or Familial Dementias):ti,ab,kw (Word variations have been searched)

3. #1 or #2

1. MeSH descriptor: [Alzheimer Disease] explode all trees
2. (Disease, Alzheimer OR Alzheimer Sclerosis OR Sclerosis, Alzheimer OR Alzheimer Syndrome OR Syndrome, Alzheimer OR Alzheimer Dementia OR Dementia, Alzheimer OR Alzheimer-Type Dementia OR Alzheimer Type Dementia OR Dementia, Alzheimer-Type OR Primary Senile Degenerative Dementia OR Dementia, Senile OR Senile Dementia OR Dementia, Alzheimer Type OR Alzheimer Type Dementia OR Senile Dementia, Alzheimer Type OR Alzheimer Type Senile Dementia OR Dementia, Primary Senile Degenerative OR Alzheimer's Disease OR Disease, Alzheimer's OR Acute Confusional Senile Dementia OR Senile Dementia, Acute Confusional OR Dementia, Presenile OR Presenile Dementia OR Alzheimer Disease, Late Onset OR Late Onset Alzheimer Disease OR Alzheimer's Disease, Focal Onset OR Focal Onset Alzheimer's Disease OR Familial Alzheimer Disease OR Alzheimer Disease, Early Onset Early Onset Alzheimer Disease OR Presenile Alzheimer Dementia) :ti,ab,kw (Word variations have been searched)

6. #4 OR #5

1. MeSH descriptor: [Drug Therapy] explode all trees
2. (Therapy, Drug or Drug Therapies or Therapies, Drug or Chemotherapy or Chemotherapies or Pharmacotherapy or Pharmacotherapies):ti,ab,kw (Word variations have been searched)

9. #7 or #8

1. MeSH descriptor: [Psychology, Social] explode all trees
2. MeSH descriptor: [Psychotherapy] explode all trees
3. (incentive*OR voucher or psychotherap* or psychosocial* or behaviour therapy or behavior therapy or reinforcement or motivation* or contingent* or advice or biofeedback or community or stimulation or education* or brief intervention or early intervention or minimal intervention or counseling or counsel* or cognitive therapy or family therapy or social skill or stress management training or supportive expressive therapy or neurobehavioral* or coping skill* or "self-control training"):ti,ab,kw (Word variations have been searched)

13. #10 or #11 or #12

1. MeSH descriptor: [Complementary Therapies] explode all trees
2. (Therapies, Complementary or Therapy, Complementary or Complementary Medicine or Medicine, Complementary or Alternative Medicine or Medicine, Alternative or Alternative Therapies or Therapies, Alternative or Therapy, Alternative):ti,ab,kw (Word variations have been searched)

16. #14 or #15

17. #9 or #13 or #16

18. #3 and #6 and #17
