# Supplementary figures and images for: Psychosocial interventions for Alzheimer’s disease cognitive symptoms: a Bayesian network meta-analysis
Source: BMC Geriatr. 2018 Aug 7;18:175. doi: 10.1186/s12877-018-0864-6 (PMC6081912; doi:10.1186/s12877-018-0864-6)

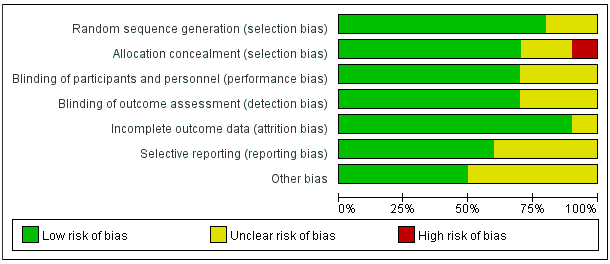


Additional file 2 Risk of Bias Graph

Supplement: Supplementary file 2 — Risk of Bias Graph. (DOCX 31 kb) [file 12877_2018_864_MOESM2_ESM.docx]

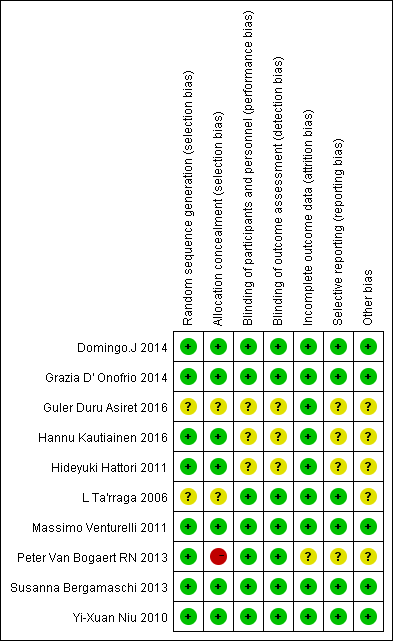


Additional file 3 Risk of Bias Summary

Supplement: Supplementary file 3 — Risk of Bias Summary. (DOCX 25 kb) [file 12877_2018_864_MOESM3_ESM.docx]

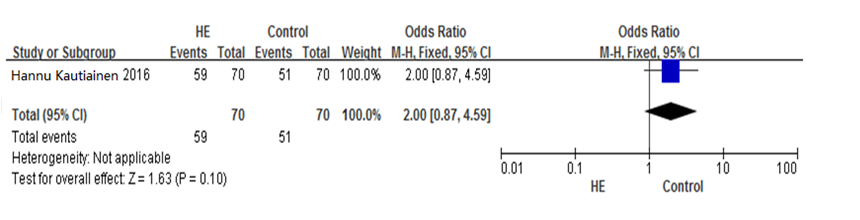

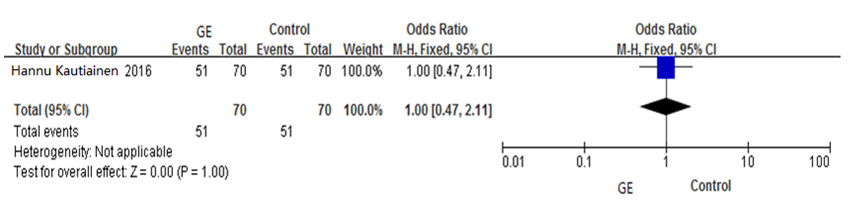

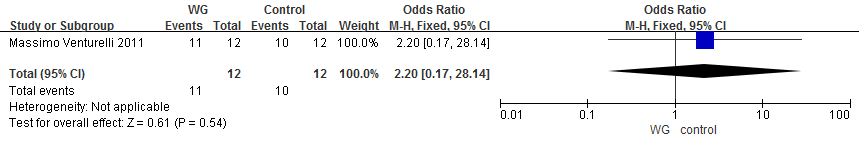


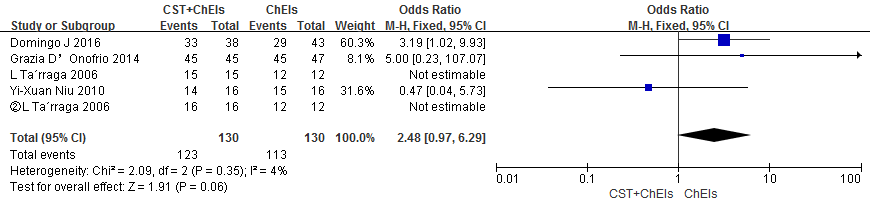

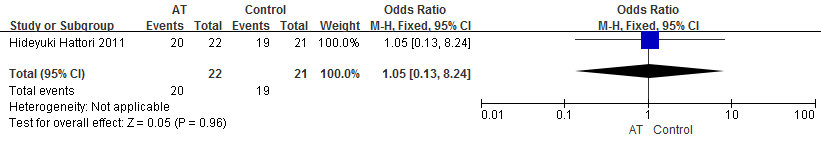


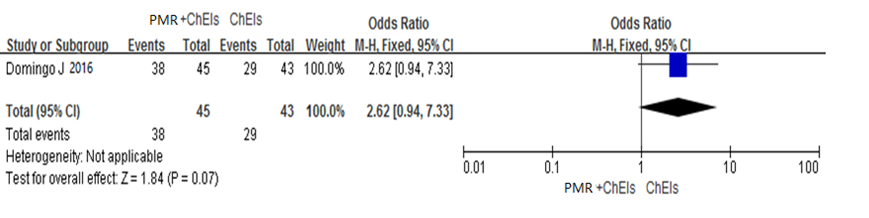


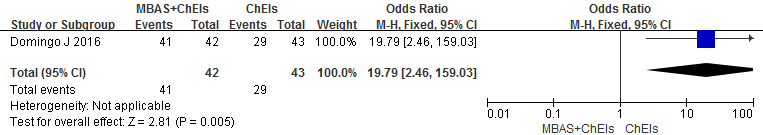


Additional file 4 Pair-wise meta-analysis of Compliance for Each Intervention

Supplement: Supplementary file 4 — Pair-wise meta-analysis of Compliance for Each Intervention. (DOCX 218 kb) [file 12877_2018_864_MOESM4_ESM.docx]
